# Targeting sFRP1 with WAY-316606 Suppresses Proliferation, Migration, and Invasion in Metastatic Melanoma

**DOI:** 10.3390/cancers18111721

**Published:** 2026-05-25

**Authors:** Dokyeong Kim, Junseong Park, Okcho Na, Dahye Nam, Sumin Cho, Minyoung Park, Songzi Zhang, Yeun-Jun Chung

**Affiliations:** 1 Precision Medicine Research Center, College of Medicine, The Catholic University of Korea, Seoul 06591, Republic of Korea; dkkim2908@catholic.ac.kr (D.K.); j.p@catholic.ac.kr (J.P.); raokcho@gmail.com (O.N.); dahae1130@catholic.ac.kr (D.N.); smcho1204@catholic.ac.kr (S.C.); m.young@catholic.ac.kr (M.P.); 2 Department of Microbiology, College of Medicine, The Catholic University of Korea, Seoul 06591, Republic of Korea; 3 Catholic Hematopoietic Stem Cell Bank, College of Medicine, The Catholic University of Korea, Seoul 06591, Republic of Korea; 4 The Medical Basic Research Innovation Center of Airway Disease in North China, Jilin University, Changchun 130021, China; zhangsz95@jlu.edu.cn; 5 Department of Medical Sciences, Graduate School of The Catholic University of Korea, Seoul 06591, Republic of Korea

**Keywords:** sFRP1, metastatic melanoma, WAY-316606, anticancer therapy

## Abstract

**Background/Objectives**: Melanoma is a highly aggressive cancer with a strong metastatic potential, and therapeutic resistance remains a major clinical challenge despite advances in targeted therapies and immunotherapies. Secreted frizzled-related protein 1 (sFRP1) exhibits context-dependent roles in cancer; however, its function in metastatic melanoma remains poorly defined. This study investigated the role of sFRP1 in melanoma progression and evaluated the anti-tumor effects of the pharmacological compound WAY-316606. **Methods**: sFRP1 expression was quantified in metastatic melanoma cell lines, xenograft models, and TCGA datasets. The anti-tumor effects of WAY-316606 on cell viability, cell cycle progression, cell migration and invasion, and expression of extracellular matrix (ECM)-related genes were assessed using WST assays, flow cytometry, wound healing and transwell invasion assays, and quantitative real-time PCR, respectively. **Results**: sFRP1 expression was consistently elevated in metastatic melanoma cell lines, xenograft models, and TCGA datasets, and high sFRP1 expression was associated with poor overall survival. WAY-316606 selectively suppressed melanoma cell viability with minimal cytotoxic effects on non-tumorigenic cells, and induced G1 phase cell cycle arrest. Furthermore, WAY-316606 markedly impaired the migratory and invasive capacities of metastatic melanoma cells, accompanied by downregulation of key ECM remodeling and fibrosis-related genes, including *VIM*, *CCN2*, *FN1*, and *TGFBI*. sFRP1 knockdown partially phenocopied the anti-migratory and gene expression effects of WAY-316606. **Conclusions**: Collectively, our findings identify sFRP1-asscoaited signaling contribute to aggressive melanoma phenotypes and highlight the therapeutic potential of its pharmacological inhibition using WAY-316606.

## Simple Summary

Melanoma is a dangerous form of skin cancer that often spreads to other parts of the body and can become resistant to current treatments. New treatment targets are therefore needed. In this study, we examined a signaling-related molecule called secreted frizzled-related protein 1 (sFRP1), whose role in metastatic melanoma has not been well understood. We found that sFRP1 levels were higher in metastatic melanoma and were linked to worse patient outcomes. Using a pharmacological inhibitor called WAY-316606, we demonstrated that blocking sFRP1 reduces melanoma cell growth, migration, and invasion while having minimal effects on normal cells. These findings suggest that sFRP1-associated signaling may contribute to metastatic melanoma and that blocking this pathway using WAY-316606 could be useful for future treatment development.

## 1. Introduction

Melanoma is a highly aggressive malignancy arising from melanocytes and is characterized by rapid progression, early dissemination, and poor prognosis in advanced stages [1,2]. Although localized melanoma can often be effectively treated by surgical excision, metastatic melanoma remains difficult to manage with high mortality [3,4]. Targeted therapies against the MAPK pathway, particularly BRAF/MEK inhibitors [5,6], as well as immune checkpoint blockade [7,8], have substantially improved clinical outcomes. However, therapeutic resistance and primary non-response remain major clinical challenges. In addition, although other targeted approaches, including EGFR-directed strategies, have also been explored in melanoma, their clinical benefit has thus far been limited [9]. These limitations underscore an unmet need to identify novel molecular targets and develop more effective therapeutic strategies for metastatic melanoma.

Among the signaling networks implicated in melanoma progression, Wnt-associated signaling has attracted attention because of its context-dependent roles in regulating cell plasticity, migration, invasion, and metastatic adaptation [10,11,12]. Secreted frizzled-related proteins (sFRPs) are a family of secreted Wnt modulators that share structural homology with the cysteine-rich domain of Frizzled (Fz) receptors but lack transmembrane and intracellular domains [13,14]. While sFRPs play important roles in embryonic development and tissue homeostasis, their functions in cancer are highly context-dependent [15]. Among them, sFRP1 has often been described as a tumor suppressor and is frequently epigenetically silenced in several epithelial malignancies [16,17,18]. However, accumulating evidence suggests that elevated sFRP1 expression is associated with tumor recurrence, invasiveness, and aggressive phenotypes in other cancer types [19,20,21]. These divergent findings highlight the need to clarify the functional role of sFRP1 specifically in metastatic melanoma.

Based on our previous single-cell RNA sequencing (scRNA-seq) analyses identifying elevated sFRP1 expression in metastatic melanoma [22], we sought to further investigate its role in melanoma progression and evaluate the therapeutic relevance of pharmacological sFRP1 inhibition. In the present study, we examined the effects of the sFRP1 inhibitor WAY-316606 on melanoma cell proliferation, migration, and invasion. Collectively, our findings identify sFRP1 may contribute to melanoma progression and support the potential therapeutic relevance of pharmacological sFRP1 inhibition in advanced melanoma.

## 2. Materials and Methods

### 2.1. Cell Culture

WM239A human melanoma cells and the related metastatic derivative lines 113/6-4L (lung), 131/4-5B1, and 131/4-5B2 (brain) [23] were maintained in RPMI-1640 medium (HyClone, Logan, UT, USA). The WM239A cell line is registered in Cellosaurus under accession number CVCL_6795. The murine melanoma lines B16F0 and B16F10 were grown in the same medium under identical culture conditions. HEK293 cells were cultured in Eagle’s Minimum Essential Medium (EMEM; Quality Biological, Gaithersburg, MD, USA), whereas Detroit 551 fibroblasts were propagated in Minimum Essential Medium (MEM; Thermo Scientific, Waltham, MA, USA). Each medium was supplemented with 10% fetal bovine serum (FBS; HyClone) and 1% penicillin-streptomycin (P/S; Sigma-Aldrich, Darmstadt, Germany). All cells were incubated at 37 °C in a humidified 5% CO_2_ atmosphere, and the medium was refreshed every 2–3 days. Routine mycoplasma screening was performed using a PCR-based detection kit (Applied Biological Materials Inc., Richmond, BC, Canada), and all cultures were confirmed to be negative.

### 2.2. Protein Extraction and Western Blot

Cells were lysed on ice in RIPA buffer (Biosesang, Seongnam, Republic of Korea) supplemented with protease and phosphatase inhibitor cocktails (GenDEPOT, Barker, TX, USA). Protein concentrations were quantified using a bovine serum albumin (BSA)-based assay (Bio-Rad, Hercules, CA, USA). For immunoblotting, 20 μg of total protein per sample was separated by SDS-PAGE and transferred to PVDF membranes (Millipore, Billerica, MA, USA). Membranes were blocked for 1.5 h at room temperature (RT) in Tris-buffered saline containing 0.1% Tween-20 (TBST) containing 5% non-fat dry milk, followed by overnight incubation at 4 °C with primary antibodies against β-actin (1:1000, Cell Signaling Technology, Boston, MA, USA) and sFRP1 (1:1000, Abcam, Cambridge, UK). After washing, membranes were incubated for 1 h at RT with HRP-conjugated anti-rabbit IgG secondary antibody (1:4000, Santa Cruz Biotechnology, Dallas, TX, USA). Protein bands were visualized using Pico PLUS or Femto chemiluminescent substrate (Thermo Scientific).

### 2.3. Mouse Xenograft Tumor Models

Xenograft models were established as previously described [24]. The animal study was designed to validate the in vivo tumorigenic relevance of the melanoma cell lines used in this study and to assess sFRP1 expression in xenograft tumor tissues, rather than to evaluate the therapeutic efficacy of WAY-316606. The experimental unit for all animal experiments was an individual mouse. Five-week-old male BALB/c nude mice (Orient Bio Laboratories, Seoul, Republic of Korea) were acclimatized for one week prior to experimentation and housed under specific pathogen-free conditions with free access to food and water. Mice were maintained under controlled temperature and humidity on a 12 h light/dark cycle in accordance with institutional guidelines. Each mouse was subcutaneously injected in the right flank with 5 × 10^5^ cells suspended in a 1:2 mixture of Matrigel (Corning, Corning, NY, USA) and serum-free RPMI medium. A total of 4–5 mice were used per melanoma cell line (WM239A, *n* = 5; 113/6-4L, *n* = 4; 131/4-5B1, *n* = 5; 131/4-5B2, *n* = 5). Sample sizes were determined based on prior experience with this xenograft model and practical considerations; no a priori sample size calculation was performed. Animals were not randomly allocated because each group corresponded to implantation of a distinct predefined melanoma cell line. No predefined inclusion or exclusion criteria were applied, and no animals or data points were excluded from the analysis. Animals were monitored daily for general health status and signs of distress. After two weeks, when tumors reached approximately 800 mm^3^, mice were euthanized by CO_2_ inhalation to minimize discomfort, and tumors were excised for immunohistochemistry (IHC) analysis. No unexpected adverse events occurred during the study. All animal procedures were conducted in accordance with institutional guidelines and were approved by the Institutional Animal Care and Use Committee of the Catholic University of Korea (CUMS-2024-0010-01; approval date: 2 January 2024). Animal experiments were reported in accordance with the ARRIVE guidelines.

### 2.4. Immunohistochemistry (IHC)

Fresh tumor tissues were fixed in 4% paraformaldehyde (PFA) for 24 h and subsequently embedded in paraffin. Sections (4 µm thick) were prepared for IHC. Slides were incubated with primary antibody against sFRP1 (1:50, Abcam), followed by incubation with an appropriate HRP-conjugated secondary antibody (ImmPRESS Goat Anti-Rabbit IgG Polymer Kit, peroxidase; Vector Laboratories, Inc., Newark, CA, USA). After washing with TBST (Biosesang), immunoreactivity was visualized using a DAB (3,3′-Diaminobenzidine) substrate solution for 1–10 min. Sections were counterstained with hematoxylin, dehydrated, and mounted. Images were acquired using a Pannoramic SCAN II slide scanner (3DHISTECH, Budapest, Hungary). Histological image assessment was performed in a blinded manner.

### 2.5. TCGA Data Analysis

Preprocessed RNA-seq data and corresponding clinical information for skin cutaneous melanoma (SKCM) patients were obtained from TCGA via the UCSC Xena Browser. All subsequent analyses were performed using R (v4.4.2). For survival analysis, patients were stratified into sFRP1_high and sFRP1_low groups based on the upper and lower quartiles of sFRP1 expression. Kaplan–Meier survival curves were generated, and statistical significance was assessed using the log-rank test. Multivariable Cox regression analysis of overall survival was performed for SFRP1 expression, age, sex, height, weight, tumor grade, Breslow depth, Clark level, and ulceration status. Hazard ratios with 95% confidence intervals and corresponding *p*-values were visualized using a forest plot.

### 2.6. Cell Cytotoxicity Assay

The effect of WAY-316606 on cell viability was evaluated using the WST-based Cell Counting Kit-8 (CCK-8; Dojindo Molecular Technologies, Kumamoto, Japan). Cells were plated in 96-well plates (SPL, Pocheon, Republic of Korea) at 8 × 10^3^ cells per well and exposed to the indicated concentrations of WAY-316606 for 72 h. After treatment, CCK-8 reagent was added to each well at 10% of the total culture volume, and plates were incubated for 1.5 h at 37 °C. Absorbance was read at 450 nm using a microplate reader (BioTek Instruments Inc., Santa Clara, CA, USA). Half-maximal inhibitory concentration (IC_50_) values were determined by nonlinear regression analysis in GraphPad Prism version 9.0.0 (GraphPad Software, Boston, MA, USA).

### 2.7. Cell Cycle Analysis

Cell cycle profiles were examined by propidium iodide (PI) staining followed by flow cytometric analysis (FACS Canto, BD Biosciences, San Jose, CA, USA). Cells were seeded in 6-well plates (SPL) at 1 × 10^5^ cells per well and incubated overnight to allow attachment. Cells were then treated with WAY-316606 (25 µM) for 18 h. The following day, cells were treated with WAY-316606 (25 µM) for 18 h. After treatment, cells were collected, fixed in 70% ethanol, and pelleted by centrifugation. Samples were then incubated with RNase A (100 µg/mL; Roche, Basel, Switzerland) for 15 min and stained with PI solution (50 µg/mL). DNA content was analyzed using FlowJo software version 10.8.1 (FlowJo LLC., Ashland, OR, USA).

### 2.8. Wound Healing Assay

For wound-healing experiments, cells were grown in 6-well plates (SPL) until they reached approximately 90% confluence. A scratch was introduced using a 200 μL pipette tip, and floating cells were removed by washing with phosphate-buffered saline (PBS; HyClone). Cells were then incubated in fresh medium with or without WAY-316606, or following siRNA transfection, as indicated for each experiment. Bright-field images were captured at the indicated time points. Wound closure was quantified using ImageJ software version 1.54r based on randomly selected fields analyzed in a blinded manner (magnification ×5), expressed as a percentage of the initial wound area, and normalized to the corresponding cell viability (%) measured at the matched assay time point.

### 2.9. Transwell Invasion Assay

Cell invasion was assessed using 8 µm pore Millicell inserts (SPL) coated with Matrigel (Corning) diluted 1:4 in serum-free RPMI medium. The inserts were placed into 24-well plates (SPL). For each insert, 2 × 10^4^ cells suspended in 100 μL of serum-free medium were added to the upper chamber, whereas the lower chamber contained 500 μL of medium supplemented with 10% FBS. After 24 h to permit cell attachment, WAY-316606 (25 μM) was applied to the upper chamber. Following 48 h of incubation, cells remaining on the upper membrane surface were removed with a cotton swab. Cells that traversed the Matrigel-coated membrane were fixed in 4% PFA and stained with 0.5% crystal violet (Sigma-Aldrich). Whole membranes were scanned using a Panoramic SCAN II system (3DHISTECH Ltd., Budapest, Hungary), and invaded cells were counted in at least three randomly selected 20× fields per insert. Invasion was expressed as a percentage of the control group after adjustment to the corresponding cell viability (%) measured at the matched assay time point.

### 2.10. siRNA Transfection

For siRNA-mediated gene silencing, cells were transfected with two independent siRNAs targeting sFRP1 or with a non-targeting control siRNA (siCtrl) using Lipofectamine RNAiMAX (Invitrogen, Carlsbad, CA, USA), according to the manufacturer’s instructions. The siRNAs were synthesized by Bioneer (Daejeon, Republic of Korea). The siRNA sequences used in this study were as follows: siSFRP1#1 forward, GAUCUAUUGGCUGAUCUAU; reverse, AUAGAUCAGCCAAUAGAUC; and siSFRP1#2 forward, CAGUUCUUCGGCUUCUACU; reverse, AGUAGAAGCCGAAGAACUG. Cells were incubated with the transfection mixture for 48 h, after which downstream assays, including qPCR, wound healing, and cell viability assays, were performed.

### 2.11. RNA Extraction and Quantitative Real Time-PCR (qRT-PCR)

Total RNA was reverse-transcribed into cDNA using the SuperScript™ VI First-Strand Synthesis System (Invitrogen) according to the manufacturer’s protocol. Quantitative real-time PCR was carried out with THUNDERBIRD™ SYBR^®^ qPCR Mix (TOYOBO, Osaka, Japan) on a CFX96 Real-Time PCR System (Bio-Rad). Primer sequences used in this study are listed in Table S2. GAPDH was used as the internal reference gene. Thermal cycling conditions were as follows: an initial denaturation step at 95 °C for 1 min, followed by 40 cycles of 95 °C for 15 s, 58–62 °C for 30 s and 72 °C for 30 s. Each reaction was run in triplicate, and relative transcript levels were calculated using the 2^−ΔΔCt^ method.

### 2.12. Statistical Analysis and Software

Statistical analyses were performed using GraphPad Prism version 9.0.0. Comparisons between two groups were conducted using Student’s *t* test, while multiple group comparisons were analyzed by one-way ANOVA with Dunnett’s post hoc test or two-way ANOVA with Bonferroni’s correction, as appropriate. Data are presented as mean ± standard error of the mean (SEM).

## 3. Results

### 3.1. Elevated sFRP1 Expression in Metastatic Melanoma

**Figure 1:**
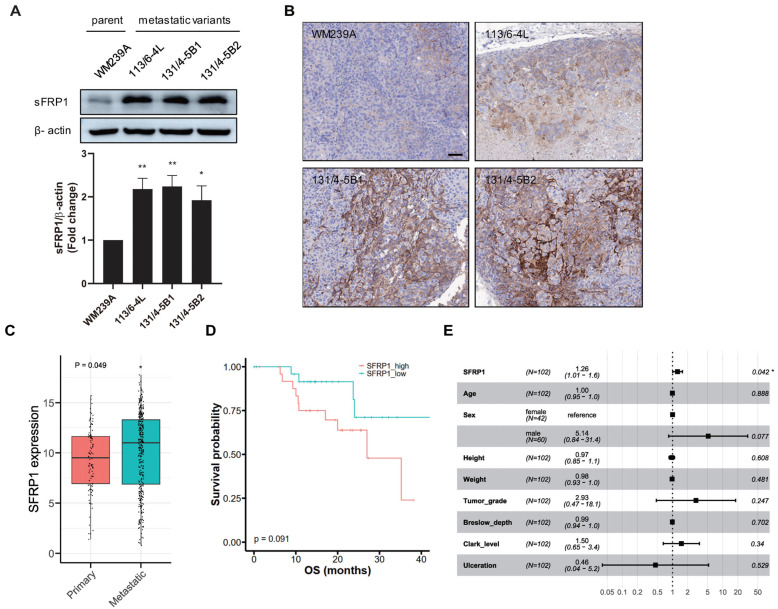
Elevated sFRP1 expression in metastatic melanoma cell lines, xenograft models, and patient datasets. (**A**) Western blot analysis of sFRP1 protein expression in parental WM239A cells and their metastatic derivative lines 113/6-4L, 131/4-5B1, and 131/4-5B2. Densitometric values were normalized to β-actin and shown relative to WM239A cells. Results are presented as mean ± SEM (*n* = 4). Statistical significance was analyzed by one-way ANOVA with Dunnett’s post hoc test (* *p* < 0.05, ** *p* < 0.01). (**B**) Representative IHC images of sFRP1 in xenograft tumors generated from the indicated melanoma cell lines. Scale bars indicate 50 µm (magnification 20×). (**C**) Expression levels of *SFRP1* in SKCM dataset obtained from TCGA. Data are shown as box plots with median values indicated; statistical significance was determined using Student’s *t*-test (* *p* < 0.05). (**D**) Kaplan–Meier survival curve of SKCM dataset from TCGA according to *SFRP1* expression levels (log-rank test). (**E**) Multivariable Cox regression analysis of overall survival in TCGA SKCM. A forest plot shows hazard ratios with 95% confidence intervals (**left**) and *p*-values (**right**) from a multivariable Cox proportional hazards model including SFRP1 expression, age, sex, height, weight, tumor grade, Breslow depth, Clark level, and ulceration status (* *p* < 0.05). The full-length uncropped western blot images of Figure 1A are shown in Figure S1.

To investigate whether sFRP1 is associated with melanoma progression, we assessed its protein levels in parental and metastatic derivatives. sFRP1 expression was markedly higher in metastatic lines (113/6-4L, 131/4-5B1, and 131/4-5B2) compared with the parental melanoma line WM239A (Figure 1A and Figure S1). Densitometric quantification confirmed significantly higher sFRP1 expression in all metastatic derivative cell lines. Consistently, xenograft models derived from these lines showed increased sFRP1 expression in metastatic tumors relative to WM239A-derived tumors, as confirmed by IHC (Figure 1B and Figure S2). To assess clinical relevance, we analyzed TCGA transcriptome data and found significantly elevated sFRP1 expression in metastatic melanoma samples compared with primary tumors (Figure 1C). Notably, patients with high sFRP1 expression considerably exhibited poorer overall survival compared with those with low expression (Figure 1D). In multivariable Cox proportional hazards analysis, SFRP1 remained an independent adverse prognostic factor (Figure 1E). Together, these results suggest that sFRP1 is associated with metastatic melanoma and may have prognostic and therapeutic relevance.

### 3.2. WAY-316606 Suppresses Melanoma Cell Viability via G1 Phase Arrest

**Figure 2:**
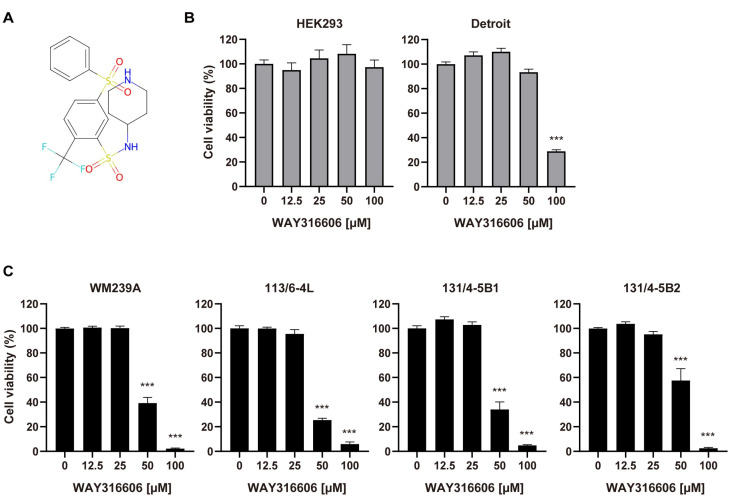
Cytotoxic effects of WAY-316606 on metastatic melanoma cell lines. (**A**) Chemical structure of WAY-316606 (WAY), retrieved from ChemSpider. (**B**) Viability of non-tumorigenic control cells, including HEK239 cells and Detroit551 cells, after 72 h exposure to the indicated concentrations of WAY-316606. Cell viability was measured using the WST assay. (**C**) Dose-dependent viability response of WM239A melanoma cells and the metastatic derivative lines 113/6-4L, 131/4-5B1, and 131/4-5B2 following 72 h treatment with WAY-316606, as determined by WST assays. Data are expressed as the means ± SEM (*n* = 6). Statistical analysis was performed using one-way ANOVA followed by Dunnett’s multiple comparisons test. Asterisks indicate significant differences relative to the untreated controls (*** *p* < 0.001).

Given the elevated sFRP1 expression in metastatic melanoma, we investigated whether its inhibition exerts antitumor effects. To provide additional context for proliferation-related outcomes, WST-based growth curves of cells used in our study were measured at 0, 24, 48, and 72 h under standard culture conditions (Figure S2). We then treated four melanoma cell lines, WM239A and its metastatic derivatives 113-6/4L, 131/4-5B1, and 131/4-5B2, with WAY-316606, a sFRP1 inhibitor (Figure 2A). To assess potential off-target toxicity, we first tested WAY-316606 in non-tumorigenic cell lines. No cytotoxicity was observed in the HEK293 human embryonic kidney cell line, while Detroit skin fibroblasts showed only mild sensitivity at concentrations exceeding 100 µM (Figure 2B). In contrast, all melanoma cell lines displayed a dose-dependent reduction in cell viability following WAY-316606 treatment (Figure 2C), with IC_50_ values summarized in Table 1. To further examine the effects of WAY_316606 in additional melanoma models, we evaluated B16F0 and B16F10 mouse melanoma cell lines (Figure S3A). In these models, WAY-316606 significantly reduced cell viability only at 100 µM, the highest concentration tested. Notably, the anti-proliferative effect of WAY-316606 was observed in both the parental WM239A cells and their metastatic derivatives, whereas its growth-inhibitory effect in B16F0 and B16F10 cells was detected only at the highest concentration tested.

**Figure 3:**
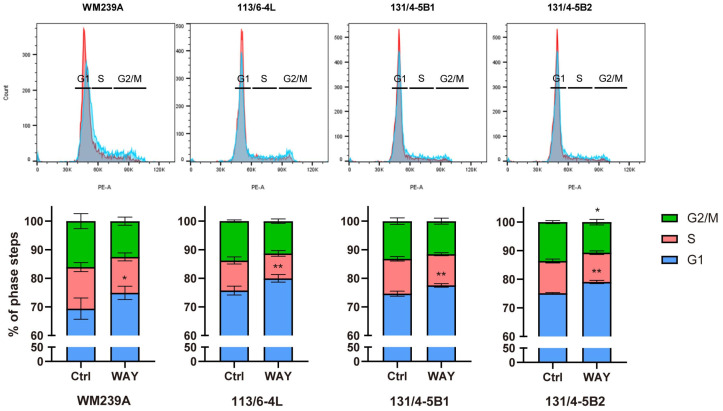
G_1_ cell cycle arrest by WAY-316606 in metastatic melanoma cell lines. Cell cycle distribution was examined by propidium iodide staining and flow cytometry after treatment with 25 μM WAY-316606 for 18 h. Upper panels show representative DNA-content histograms are shown, while lower panels present quantitative summaries of cell-cycle phase distribution. For the histograms (upper panels), the blue line represents the control group, whereas the red line represents WAY-316606-treated cells. Data are expressed as mean ± SEM (*n* = 4). Statistical significance was assessed using two-way ANOVA with Bonferroni’s multiple comparisons test, with comparisons to the control group (* *p* < 0.05, ** *p* < 0.01).

For subsequent functional assays, we used 25 µM WAY-316606, a concentration below the IC_50_ values in all melanoma cell lines (Figure 2C and Table 1). To determine whether the observed growth inhibition was associated with cell cycle arrest, we performed PI staining followed by flow cytometry. WAY-316606 treatment led to a significant accumulation of melanoma cells in the G1 phase, indicative of cell cycle arrest (Figure 3). Collectively, these results demonstrate that WAY-316606 suppresses melanoma cell viability by inducing G1 phase cell cycle arrest.

### 3.3. WAY-316606 Impairs Migration and Invasion and Alters ECM Remodeling—And Wnt-Related Gene Expression in Melanoma Cells

**Figure 4:**
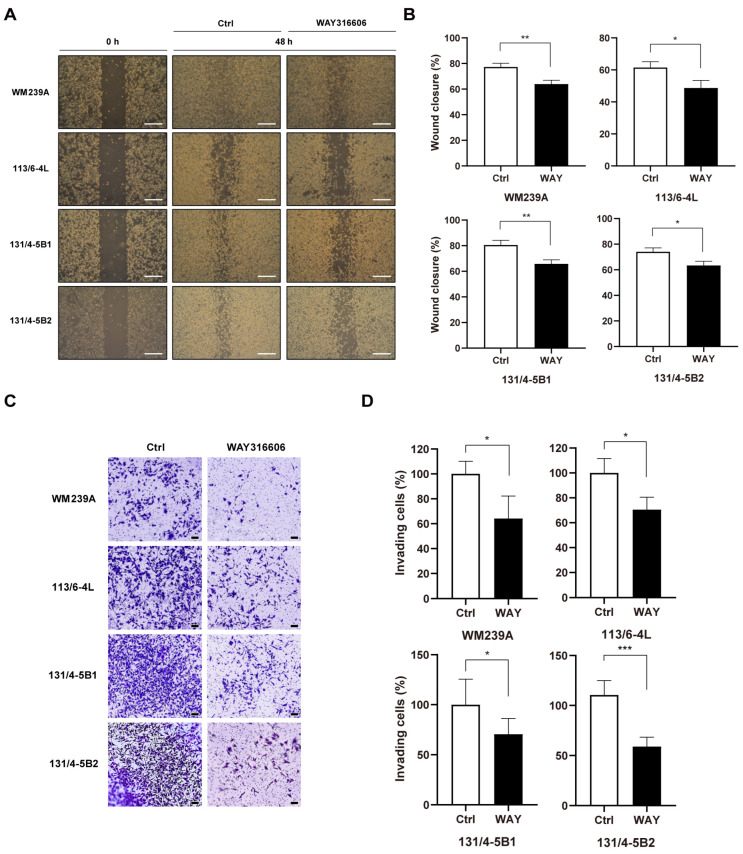
Inhibition of migratory and invasive properties by WAY-316606 in metastatic melanoma cells. (**A**) Wound healing assay illustrating the effect of WAY-316606 (25 μM) on cell migration at 48 h post-scratch. Scale bars indicate 500 µm (magnification 5×). (**B**) Bar graph showing the percentage of wound closure relative to the control group. Quantification was performed using randomly selected fields analyzed in a blinded manner. Migration values were normalized to the corresponding cell viability (%) measured at the matched assay time point. Data are presented as mean ± SEM (*n* = 3). (**C**) Transwell invasion assay demonstrating a significant reduction in the number of invaded cells following WAY-316606 (25 μM) treatment. Scale bars represent 50 µm (magnification 20×). (**D**) Bar graphs representing invaded cells quantified from at least three randomly selected fields per insert (one membrane/insert per well), analyzed in a blinded manner. Data are presented as mean ± SEM (*n* = 3). Statistical significance was determined using an unpaired Student’s *t*-test (* *p* < 0.05, ** *p* < 0.01, *** *p* < 0.001).

To assess the anti-metastatic potential of WAY-316606, we performed wound-healing and transwell invasion assays in the parental WM239A cells and their metastatic derivatives. At 48 h post-scratch, untreated cells efficiently closed the wound area, whereas WAY-316606-treated cells showed significantly impaired migration, with a persistently wider wound gap (Figure 4A,B). Similarly, transwell invasion assays revealed a marked reduction in invasion (Figure 4C,D). In parallel, we also evaluated the anti-migratory effect of WAY-316606 in B16F0 and F10 mouse melanoma cells. In these models, WAY-316606 suppressed cell migration in both cell lines (Figure S3B,C).

**Figure 5:**
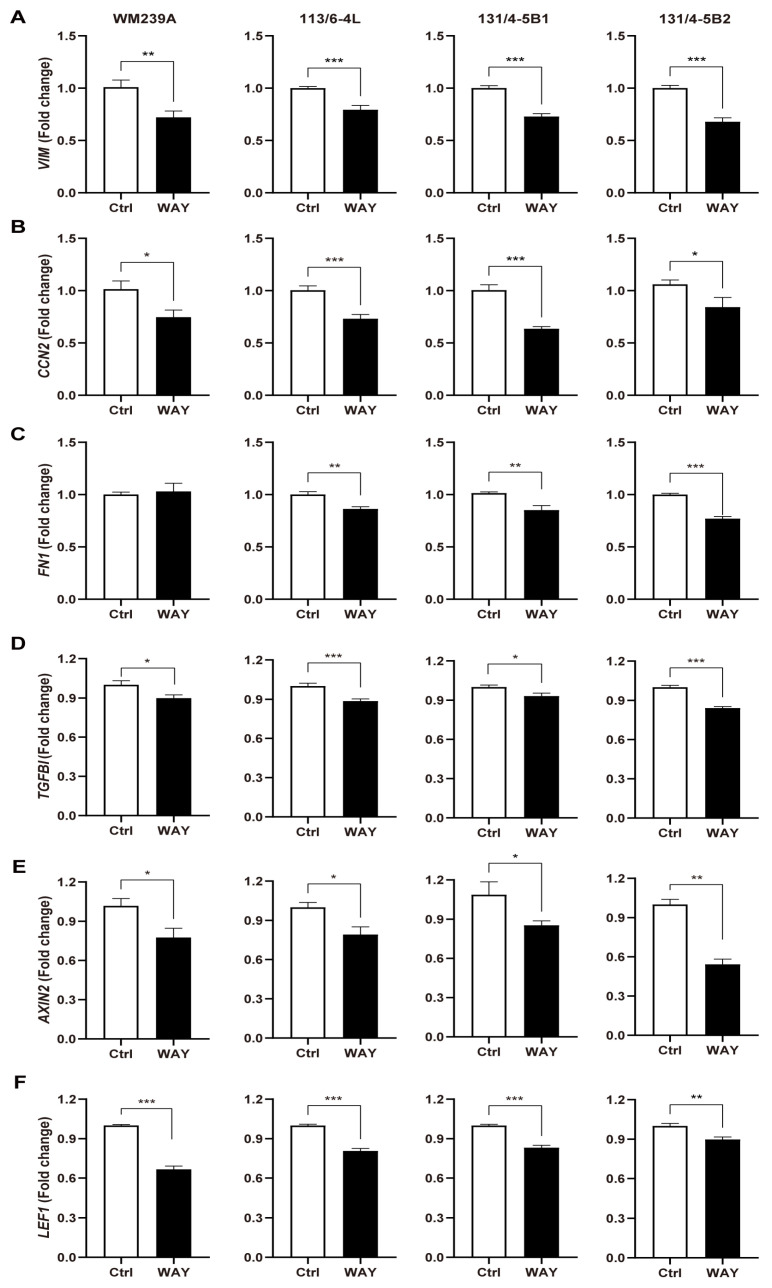
WAY-316606 alters ECM remodeling—and Wnt-related gene expression in melanoma cells. (**A**–**F**) qPCR analysis of *VIM* (**A**), *CCN2* (**B**), *FN1* (**C**), *TGFBI* (**D**), *AXIN2* (**E**), and *LEF1* (**F**) expression in WM239A cells and their metastatic derivatives (113/6-4L, 131/4-5B1, and 131/4-5B2) following treatment with WAY-316606 25 µM. Gene expression levels are presented as fold change relative to the control group. Data are shown as mean ± SEM (*n* = 3). Statistical significance was determined using an unpaired Student’s *t*-test (* *p* < 0.05, ** *p* < 0.01, *** *p* < 0.001).

Because metastatic dissemination is closely linked to extracellular matrix (ECM) remodeling, we next examined the expression of fibrosis- and ECM-associated genes following WAY-316606 treatment. qPCR analysis showed that WAY-316606 treatment consistently reduced the expression of *VIM*, *CCN2*, *FN1*, and *TGFBI* across all four melanoma cell lines (Figure 5A–D). We further assessed Wnt-associated genes and found that *AXIN2* and *LEF1* expression were both reduced across all four melanoma cell lines following WAY-316606 treatment (Figure 5E,F). To provide orthogonal support for these pharmacological findings, we additionally silenced sFRP1 using two independent siRNAs (Figure S4A). sFRP1 knockdown significantly reduced wound closure in 131/4-5B1 and 131/4-5B2 cells (Figure S4B,C). The expression of *FN1*, *TGFBI*, and *LEF1* was reduced in most tested cell lines following sFRP1 knockdown (Figure S5A–C).

To minimize the possibility that reduced migration and invasion were secondary to cytotoxicity, all migration and invasion data were normalized to cell viability measured at the corresponding assay-matched time points. Under these conditions, neither WAY-316606 nor sFRP1 knockdown caused substantial loss of viability (Table S1). Collectively, these findings indicate that WAY-316606 suppresses melanoma cell motility and invasion under non-cytotoxic assay conditions and is associated with altered ECM remodeling– and Wnt-related gene expression. Moreover, the reduction in migration and ECM/Wnt-related gene expression observed after sFRP1 knockdown supports the involvement of sFRP1-associated signaling.

## 4. Discussion

In this study, we first identified that sFRP1 expression is markedly elevated in metastatic melanoma, and its pharmacological inhibition suppresses melanoma cell viability, migration, and invasion. Collectively, these findings suggest that sFRP1-associated signaling may contribute to aggressive melanoma phenotypes in this model system. sFRP1 has been traditionally classified as a tumor suppressor due to its frequent loss of expression in several epithelial malignancies, including colorectal [16,25], prostate [26], renal [27], and head and neck cancers [28]. In these tumor types, sFRP1 silencing is commonly associated with promoter hypermethylation and transcriptional repression, supporting its canonical role as a negative regulator of tumor initiation and progression [16,17,18]. Conversely, accumulating evidence indicates that sFRP1 can exert pro-tumorigenic functions in basal-like breast [19], liver [20], and gastric cancer [21], where its upregulation promotes epithelial–mesenchymal transition (EMT) and invasive behavior through activation of pathways such as TGF-β/Smad3 signaling or FGFR2-HIF axis. Together, these divergent findings underscore the highly context-dependent functions of sFRP1 across cancer types [15,29]. This context dependency has important implications for therapeutic strategies targeting sFRP1, as the biological consequences of its inhibition are likely to vary depending on tumor type and signaling context. In metastatic melanoma, our data support that sFRP1 is more likely to function as a contributory factor associated with tumor progression rather than as a canonical tumor suppressor. At the same time, the moderate magnitude of some inhibitory effects suggests that sFRP1 is unlikely to act as a sole driver of melanoma aggressiveness but rather may function in concert with other molecular pathways.

Functionally, pharmacologic inhibition of sFRP1 with WAY-316606 effectively reduced melanoma cell viability and induced G1-phase cell cycle arrest. These results are consistent with the well-established roles of Wnt/β-catenin pathway modulators in controlling cell cycle progression [30,31]. Consistent with these, our findings also show a potential connection between sFRP1-associated signaling and canonical Wnt-related transcriptional output. WAY-316606 reduced *AXIN2* and *LEF1* expression, and *LEF1* was also decreased following sFRP1 knockdown. These observations provide mechanistic support for the idea that sFRP1-associated signaling may intersect with Wnt-related pathways in metastatic melanoma. However, the current data does not establish canonical Wnt signaling as the sole or dominant downstream mechanism, and the relationship between sFRP1 and Wnt signaling in this setting likely remains context dependent. An important consideration is the discrepancy between the reported biochemical potency of WAY-316606 and the higher concentrations required to elicit cellular phenotypes in melanoma cells. Biochemical IC_50_ values are typically measured under simplified cell-free conditions, whereas cellular responses may be influenced by effective extracellular availability, protein binding, compound stability, and pathway context [32,33]. In addition, sFRP1 functions as a secreted extracellular modulator rather than a direct intracellular regulator of cell survival, which may explain why cellular phenotypes do not scale proportionally with biochemical inhibition. In addition to its effects on cell viability and G1-phase cell cycle arrest, WAY-316606 significantly impaired the migratory and invasive capacities of metastatic melanoma cells under assay conditions in which substantial cytotoxicity was not observed. These phenotypic effects were accompanied by downregulation of ECM and fibrosis-related genes, including *VIM, CCN2*, *FN1*, and *TGFBI*. These genes are well-established mediators of ECM remodeling and metastatic niche formation [34,35,36,37], and prior studies have implicated sFRP1 activity within the tumor microenvironment in promoting aggressive tumor phenotypes [20,38]. In additional melanoma models, WAY-316606 also reduced migration in B16F0 and B16F10 mouse melanoma cells under non-cytotoxic assay conditions. Moreover, genetic silencing of sFRP1 reduced migration in 131/4-5B1 and 131/4-5B2 cells and decreased ECM/Wnt-related gene expression. Although the effects of WAY-316606 treatment and sFRP1 knockdown were not completely concordant, these findings support the involvement of sFRP1-associated signaling rather than nonspecific pharmacological effects alone. Collectively, our findings support the possibility that sFRP1-associated signaling contributes to melanoma dissemination, at least in part, through ECM reorganization and highlight the therapeutic potential of WAY-316606 in metastatic melanoma.

Current standard-of-care therapies, including BRAF/MEK inhibitors and immune checkpoint blockade, have significantly extended patient survival, yet resistance and non-response remain prevalent [6,7,8]. Using scRNA-seq, we previously identified elevated *CCND1* expression in distant metastatic melanoma cell populations and demonstrated that pharmacological inhibition of *CCND1* with arcyriaflavin A exerts anti-tumor effects in melanoma cells [24]. Building on these findings, we evaluated combinatorial targeting of *CCND1* and *SFRP1* using arcyriaflavin A and WAY-316606, respectively. Although each agent independently suppressed melanoma cell growth, their combined treatment resulted in limited synergistic effects. One plausible explanation is that arcyriaflavin A and WAY-316606 converge on overlapping proliferative signaling pathways, particularly those regulating cell cycle progression, thereby constraining additive or synergistic efficacy. Despite the limited synergy observed with arcyriaflavin A, targeting sFRP1 may represent a complementary therapeutic strategy, either as monotherapy or in combination with established regimens, including BRAF/MEK inhibitors or immune checkpoint blockade. Several limitations warrant consideration. First, the therapeutic efficacy of WAY-316606 was not evaluated in tumor-bearing mice, and its translational relevance remains to be established in an in vivo treatment setting. Given the substantial gap between biochemical and cellular potency, future studies will require careful optimization of dosing regimen, route of administration, pharmacokinetics, and tolerability before therapeutic evaluation in vivo. Second, although genetic knockdown provided orthogonal support for the role of sFRP1-associated signaling, the pharmacological and genetic perturbations did not fully overlap across all cell lines. In addition, definitive rescue-based validation was not established in the current study. Therefore, the effects of WAY-316606 should not be interpreted as conclusively on-target, and direct target specificity remains to be further validated. Third, broader validation in genetically diverse human melanoma models is still needed, as B16F0 and B16F10 cells only partially supported generalizability. Finally, non-tumorigenic controls were used instead of normal melanocytes, and future studies will be required to better define melanoma-specific vulnerability to sFRP1 inhibition. In addition, because Detroit551 fibroblasts showed cytotoxicity at the highest concentration tested, the apparent selectivity of WAY-316606 should be interpreted cautiously, particularly at higher concentrations.

## 5. Conclusions

**Figure 6:**
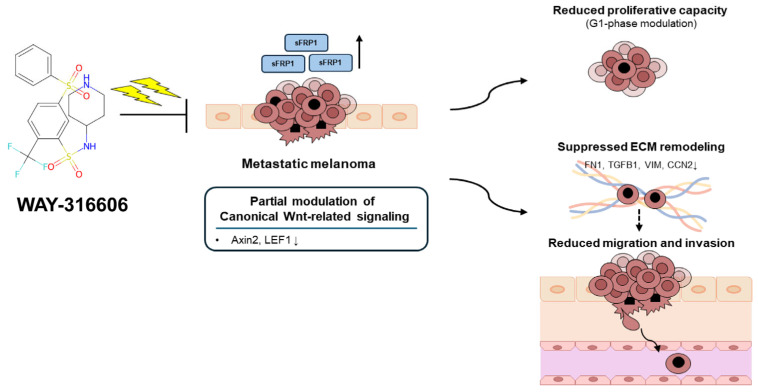
Proposed model of WAY-316606 action in metastatic melanoma. A schematic summary of the principal findings of this study, illustrating that WAY-316606 suppresses proliferative and invasive melanoma phenotypes and is associated with partial modulation of sFRP1-related and canonical Wnt-related signaling.

This study suggests that sFRP1 may contribute to metastatic melanoma progression and demonstrates that pharmacological inhibition with WAY-316606 attenuates aggressive phenotypes, at least in part, through modulation of ECM remodeling, cell cycle progression, and canonical Wnt-related signaling (Figure 6). Although further mechanistic and in vivo validation is required, these findings support the potential therapeutic relevance of targeting sFRP1-associated signaling in advanced melanoma.

## Figures and Tables

**Table 1:** IC_50_ by WAY-316606 in melanoma cell lines.

Cell Line	IC50 [μM] (±S.D)
	72 h
WM239A	47.07 (±8.40)
113/6-4L	40.47 (±7.80)
131/4-5B1	42.77 (±11.48)
131/4-5B2	60.91 (±16.33)

## Data Availability

The data generated in the present study may be requested from the corresponding author. The R scripts underlying the TCGA analyses have been deposited in Zenodo (DOI: 10.5281/zenodo.20005349).

## Supplementary Materials

The following supporting information can be downloaded at: https://www.mdpi.com/article/10.3390/cancers18111721/s1, Figure S1: Full-length uncropped western blot images corresponding to Figure 1A; Figure S2: Growth characteristics of melanoma cell lines; Figure S3: WAY-316606 reduces cell viability and migration in B16F0 and B16F10 melanoma cells; Figure S4: Silencing of sFRP1 suppresses migration in metastatic melanoma cell lines; Figure S5: Silencing of sFRP1 modulates ECM-related and Wnt-associated gene expression in melanoma cell lines; Table S1: Cell viability at assay-matched time points used for migration and invasion experiments; Table S2: The list of primer used for qRT-PCR.

## Abbreviations

The following abbreviations are used in this manuscript:

BSA	bovine serum albumin
CCK	cell counting kit
DAB	3,3′-Diaminobenzidine
cDNA	complementary deoxyribonucleic acid
ECM	extracellular matrix
EMEM	Eagle’s Minimum Essential Medium
EMT	epithelial–mesenchymal transition
FBS	fetal bovine serum
GAPDH	glyceraldehyde 3-phosphate dehydrogenase
HRP	horseradish peroxidase
IHC	immunohistochemistry
MEM	Minimum Essential Medium
PBS	phosphate-buffered saline
PFA	paraformaldehyde
PI	propidium iodide
RT	room temperature
scRNA-seq	single-cell RNA sequencing
sFRP	Secreted frizzed-related protein
SKCM	Skin cutaneous melanoma
TBS	Tris-buffered saline
TGF-β	Transforming growth factor-beta
