# Supplementary material for: Targeting sFRP1 with WAY-316606 Suppresses Proliferation, Migration, and Invasion in Metastatic Melanoma
Source: Cancers (Basel). 2026 May 25;18(11):1721. doi: 10.3390/cancers18111721 (PMC13255980; doi:10.3390/cancers18111721)
Supplement: Supplementary file 1 [file cancers-18-01721-s001.zip › cancers-4205296-supplementary.pdf]

Supplementary Material

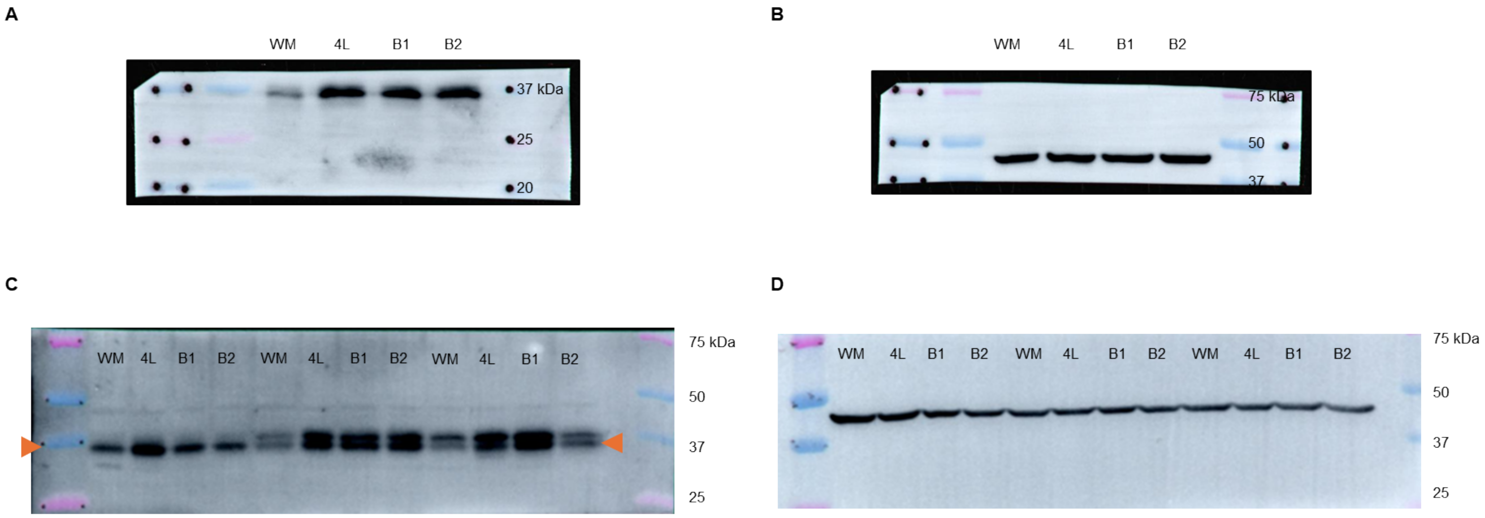

Figure S1. Full-length uncropped western blot images corresponding to Figures 1A. (**A–D**) Full-length uncropped western blot images corresponding to Figure 1A, showing sFRP1 and β-actin expression in WM239A cells and their metastatic derivatives (113/6-4L, 131/4-5B1, and 131/4-5B2). The original full-length images of sFRP1 (**A**) and β-actin (**B**) are shown, and the full-length uncropped western blot images of sFRP1 (**C**) and β-actin (**D**) used for quantification in Figure 1A are provided.

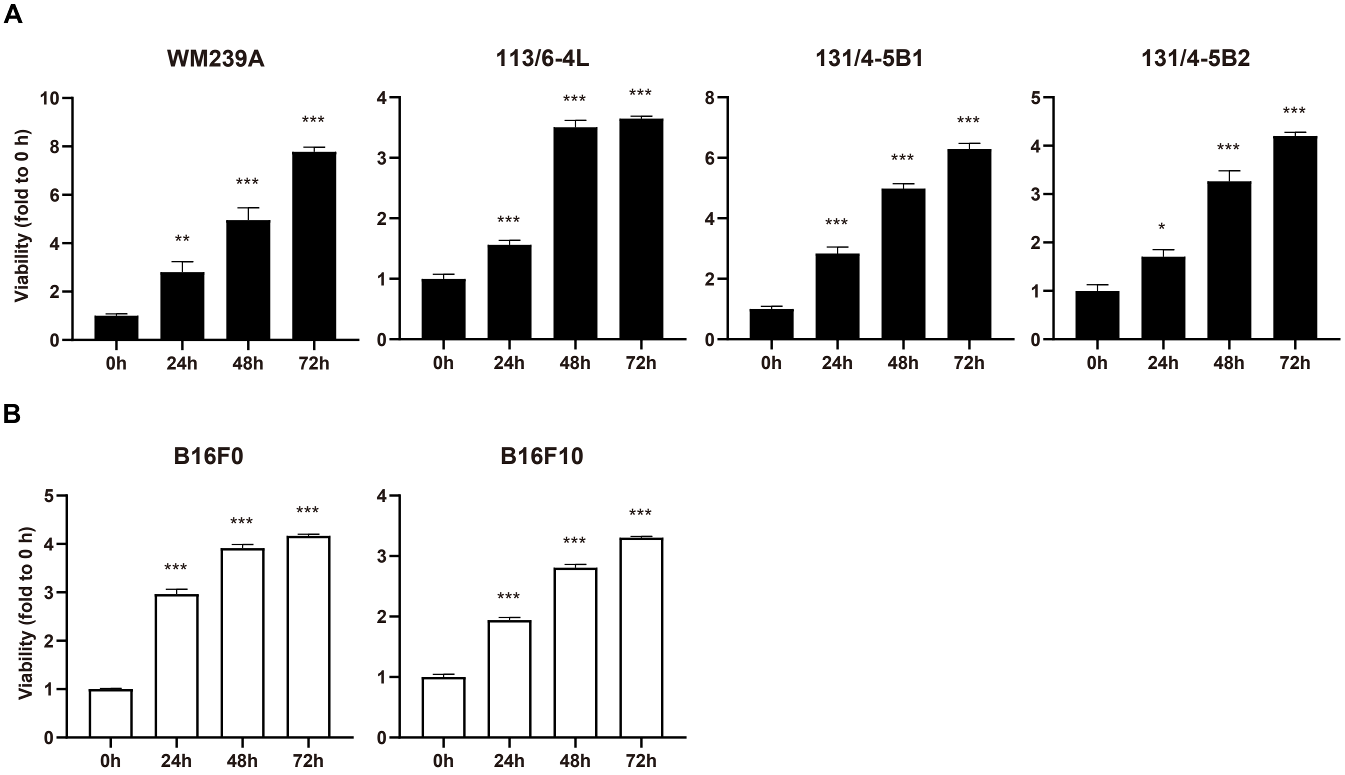

Figure S2. Growth characteristics of melanoma cell lines. (**A**) WST-based growth curves of WM239A cells and their metastatic derivatives (113/6-4L, 131/4-5B1, and 131/4-5B2), and (**B**) B16F0 and B16F10 cells measured at 0, 24, 48, and 72 h under standard culture conditions. Viability is expressed as fold change relative to 0 h. Data are shown as mean ± SEM (n = 5). Statistical significance was determined using one-way ANOVA followed by Dunnett's multiple comparisons test, with each time point compared to 0 h (*$P < 0.05$, **$P < 0.01$, ***$P < 0.001$).

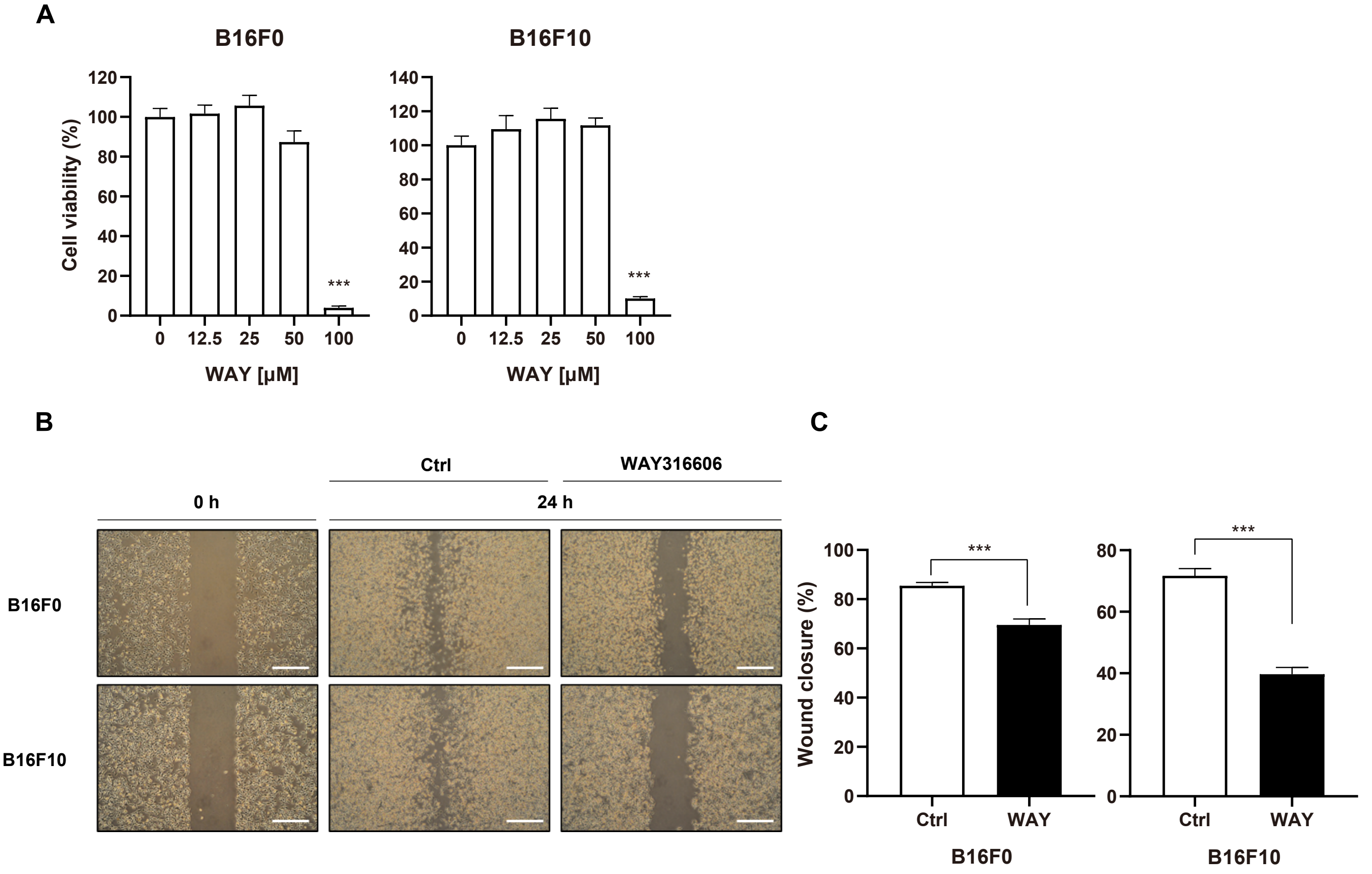

Figure S3. WAY-316606 reduces cell viability and migration in B16F0 and B16F10 melanoma cells. (**A**) Cell viability of B16F0 and B16F10 cells following treatment with WAY-316606 at the indicated concentrations for 72 h, as determined by WST assay. Data are presented as mean ± SEM (n = 6). Statistical significance was determined using one-way ANOVA followed by Dunnett's multiple comparisons test, with each treatment group compared to control (***$P < 0.001$). (**B**) Representative images of wound-healing assays in B16F0 and B16F10 cells treated with WAY-316606. Images were captured at 0 h and 24 h after scratch formation. Scale bars, 500 μm (magnification 5×). (**C**) Quantification of wound closure in B16F0 and B16F10 cells following WAY-316606 50 μM treatment. Wound closure is presented as a percentage of the initial wound area. Three scratch lines were made per well, and at least two random fields were analyzed per line (minimum six fields per well) using blinded field selection. Data are presented as mean ± SEM from n = 5 wells per group. Statistical significance was determined using an unpaired Student's t-test (***$P < 0.001$).

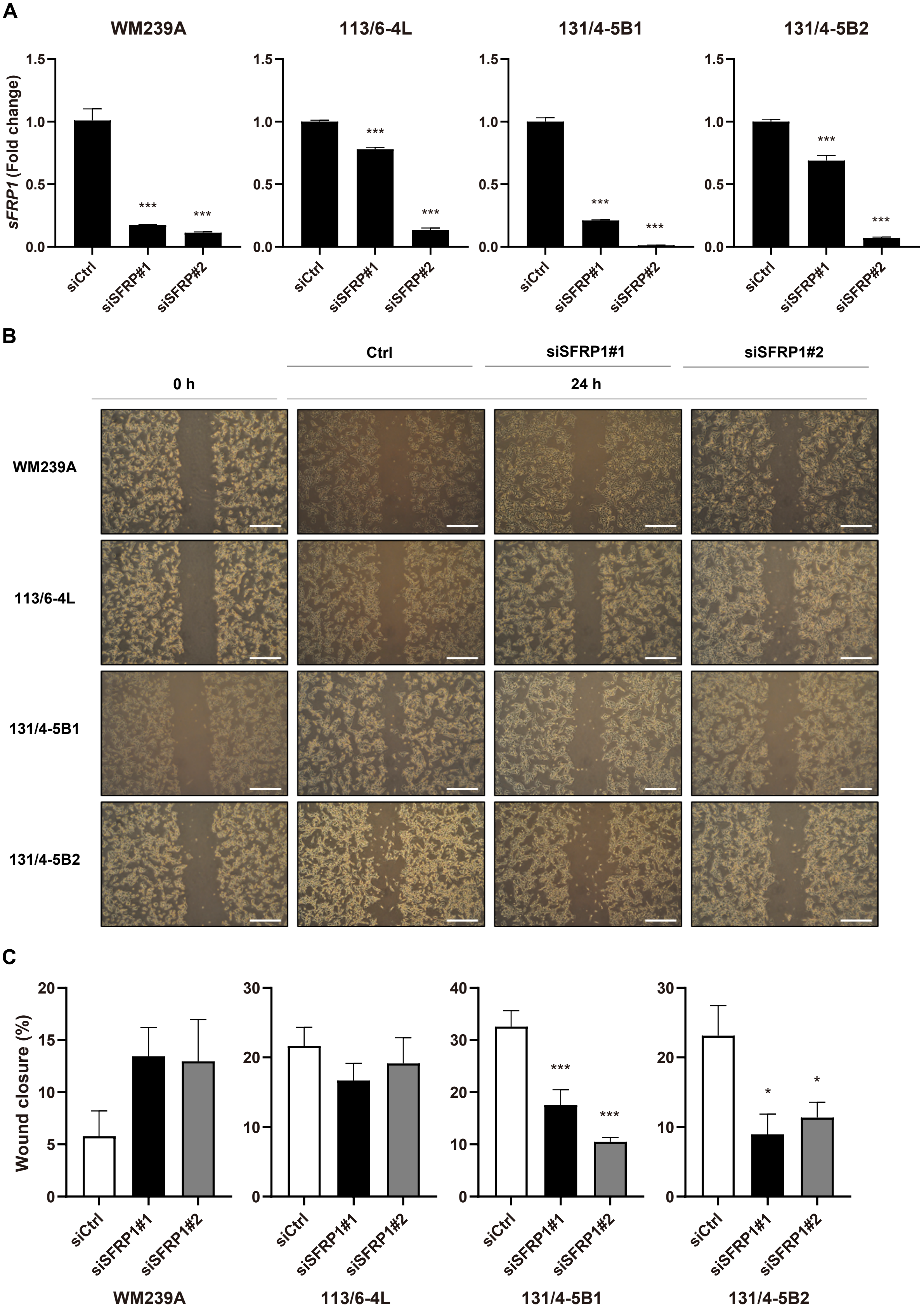
A
WM239A
113/6-4L
131/4-5B1
131/4-5B2
sFRP1 (Fold change)
1.5
1.0
0.5
0.0
***
siCtrl
siSFRP#1
siSFRP#2
B
0 h
Ctrl
siSFRP1#1
siSFRP1#2
24 h
WM239A
113/6-4L
131/4-5B1
131/4-5B2
C
Wound closure (%)
*
siCtrl
siSFRP1#1
siSFRP1#2
WM239A
113/6-4L
131/4-5B1
131/4-5B2

Figure S4. Silencing of sFRP1 suppresses migration in metastatic melanoma cell lines. (**A**) Knockdown efficiency of sFRP1 following transfection with two independent siRNAs (siSFRP1#1 and siSFRP1#2) in WM239A cells and their metastatic derivatives (113/6-4L, 131/4-5B1, and 131/4-5B2), as determined by qPCR. Expression levels are presented as fold change relative to the siCtrl group. (**B**) Representative images of wound-healing assays performed in WM239A cells and their metastatic derivatives following transfection with siCtrl, siSFRP1#1, or siSFRP1#2. Images were captured at 0 h and 24 h after scratch formation. Scale bar, 500 µm (magnification 5×). (**C**) Quantification of wound closure in WM239A cells and their metastatic derivatives after transfection with siCtrl, siSFRP1#1, or siSFRP1#2. For each well, one scratch line was generated, and two random fields were analyzed using blinded field selection. Data are presented as mean ± SEM ($n \geq 6$). Statistical significance was determined using one-way ANOVA followed by Dunnett's multiple comparisons test, with each siSFRP1 group compared to siCtrl (*$P < 0.05$, ***$P < 0.001$).

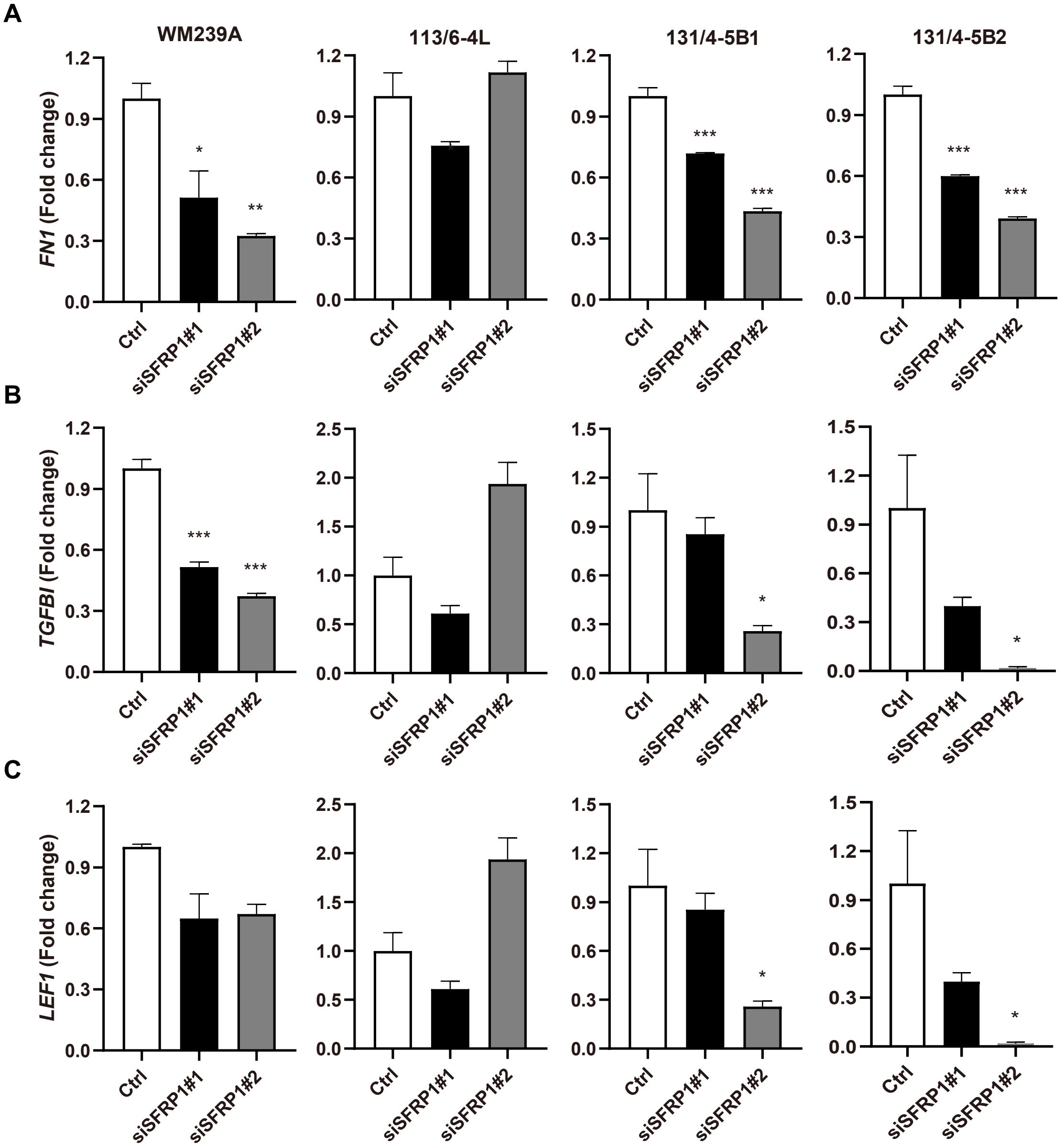

Figure S5. Silencing of sFRP1 modulates ECM-related and Wnt-associated gene expression in melanoma cell lines. qPCR analysis of (**A**) *FN1* and (**B**) *TGFBI*, and (**C**) *LEF1* in WM239A cells and their metastatic derivatives (113/6-4L, 131/4-5B1, and 131/4-5B2) following transfection with siCtrl, siSFRP1#1, or siSFRP1#2. Gene expression levels are presented as fold change relative to the siCtrl group. Data are shown as mean ± SEM (n = 3). Statistical significance was determined using one-way ANOVA followed by Dunnett's multiple comparisons test, with each siSFRP1 group compared with siCtrl (*$P < 0.05$, **$P < 0.01$, ***$P < 0.001$).

Table S1. **Cell viability at assay-matched time points used for migration and invasion experiments**

	Cell viability (%, relative to control)		
	WAY-316606 (48 h)	siSFRP1#1 (24 h)	siSFRP1#2 (24 h)
WM239A	105.7	102.8	104.1
113/6-4L	105.5	103.2	117.6
131/4-5B1	108.5	123.8	157.9
131/4-5B2	101.8	90.2	90.8
	WAY-316606 (24 h)		
B16F0	92.3		
B16F10	112.4		

Table S2 The list of primer used for qRT-PCR

Gene	F/R	Sequence
VIM	Forward	GTTTCCCCTAAACCGCTAGG
	Reverse	AGCGAGAGTGGCAGAGGA
CCN2	Forward	ATCTTCGGTGGTACGGTGTA
	Reverse	ACGTGTCTTCCAGTCGGTAA
TGFBI	Forward	TACCTGAACCCGTGTTGCTCTC
	Reverse	GTTGCTGAGGTATCGCCAGGAA
FN1	Forward	ACAACACCGAGGTGACTGAGAC
	Reverse	GGACACAACGATGCTTCCTGAG
AXIN2	Forward	ACCATTCGCAGTACCACTCC
	Reverse	CGATCTCCTCCTTGGTCTTG
LEF1	Forward	CGAATGTCGTTGCTGAGTGT
	Reverse	CAGACCAGCCTGGATAAAGC
SFRP1	Forward	CAGTTCTTCGGCTTCTACTGG
	Reverse	TTTCAACTCGTTGTCACAGGG
GAPDH	Forward	GGTGGAGGTCGGGAGTCAACGGA
	Reverse	GAGGGATCTCGCTCCTGGAGGA
